# Dr. Silas Hubbard

**Published:** 1917-06

**Authors:** 


**Dr. Silas Hubbard,** Medical College of Castleton, Vt. (extinct since 1861) 1841, of E. Aurora, died at the home of his daughter in Buffalo, May 18, aged 96. He was born in Mayville, Chautauqua Co., May 9, 1821, and came to Buffalo with his parents, when 9 years old. His education was received at the Lima Seminary, E. Aurora Academy and Allegheny College. He was a practitioner in Buffalo from 1842 to 1855, when he went to McLean Co., Ill., practicing first in Bloomington and Hudson. In 1900, he returned to Buffalo but lived for most of the time in E. Aurora. He contributed several articles to the first series of the Buffalo Medical Journal and has shown his continued interest in medical matters by quite recent though brief communications. So far as known, he was, at the time of his death, the oldest physician in the state.

